# Alginate-Based Encapsulation Fabrication Technique for Drug Delivery: An Updated Review of Particle Type, Formulation Technique, Pharmaceutical Ingredient, and Targeted Delivery System

**DOI:** 10.3390/pharmaceutics16030370

**Published:** 2024-03-06

**Authors:** Joanne Lai, Abul Kalam Azad, Wan Mohd Azizi Wan Sulaiman, Vinoth Kumarasamy, Vetriselvan Subramaniyan, Salah Abdalrazak Alshehade

**Affiliations:** 1 Faculty of Pharmacy, MAHSA University, Jenjarom 42610, Selangor, Malaysia; joanne121700@gmail.com (J.L.); drwanazizi@ucmi.edu.my (W.M.A.W.S.); salah_alsh@outlook.com (S.A.A.); 2 Faculty of Pharmacy, University College of MAIWP International, Kuala Lumpur 68100, Selangor, Malaysia; 3 Department of Parasitology and Medical Entomology, Faculty of Medicine, Universiti Kebangsaan Malaysia, Jalan Yaacob Latif, Kuala Lumpur 56000, Selangor, Malaysia; 4 Pharmacology Unit, Jeffrey Cheah School of Medicine and Health Sciences, Monash University, Jalan Lagoon Selatan, Bandar Sunway 47500, Selangor, Malaysia; 5 Center for Transdisciplinary Research, Department of Pharmacology, Saveetha Dental College, Saveetha Institute of Medical and Technical Sciences, Saveetha University, Chennai 600077, Tamil Nadu, India; subramaniyan.vetriselvan@monash.edu

**Keywords:** alginate, microparticles, encapsulation, nanoparticles, microgels, targeted delivery

## Abstract

Alginate is a natural biopolymer widely studied for pharmaceutical applications due to its biocompatibility, low toxicity, and mild gelation abilities. This review summarizes recent advances in alginate-based encapsulation systems for targeted drug delivery. Alginate formulations like microparticles, nanoparticles, microgels, and composites fabricated by methods including ionic gelation, emulsification, spray drying, and freeze drying enable tailored drug loading, enhanced stability, and sustained release kinetics. Alginate microspheres prepared by spray drying or ionic gelation provide gastric protection and colon-targeted release of orally delivered drugs. Alginate nanoparticles exhibit enhanced cellular uptake and tumor-targeting capabilities through the enhanced permeation and retention effect. Crosslinked alginate microgels allow high drug loading and controlled release profiles. Composite alginate gels with cellulose, chitosan, or inorganic nanomaterials display improved mechanical properties, mucoadhesion, and tunable release kinetics. Alginate-based wound dressings containing antimicrobial nanoparticles promote healing of burns and chronic wounds through sustained topical delivery. Although alginate is well-established as a pharmaceutical excipient, more extensive in vivo testing is needed to assess clinical safety and efficacy of emerging formulations prior to human trials. Future opportunities include engineered systems combining stimuli-responsiveness, active targeting, and diagnostic capabilities. In summary, this review discusses recent advances in alginate encapsulation techniques for oral, transdermal, and intravenous delivery, with an emphasis on approaches enabling targeted and sustained drug release for enhanced therapeutic outcomes.

## 1. Introduction

Alginate, a natural anionic polysaccharide derived from brown algae, has become a widely used biomaterial in pharmaceutical research for the development of drug delivery systems. The ability of alginate to form hydrogels through ionic crosslinking with divalent cations such as Ca^2+^ makes it ideal for encapsulating bioactive molecules. In recent years, there has been growing interest in alginate encapsulation technology for oral, topical, and targeted drug delivery [1]. Marine brown algae, which is under the class of Phaeophyceae, is the natural source of this polyanionic hetero-polysaccharide polymer [2,3,4]. It is generally derived from three main species, including *Macrocystis pyrifera*, *Laminaria digitata*, and *Laminaria saccharina* [5,6]. Alginate is an unbranched copolymer that consists of two monomers, including a β-(1→4)-linked D-mannuronic acid (M block) and an α-(1→4)-linked L-guluronic acid (G block), as stated by Azad et al. [7]. Blocks can be arranged with different proportions of MM, GG, and MG blocks, as well as with different positions in the chain and in different numbers, hence making various arrangements such as MMMM and GMGM [1].

Through electrostatic, covalent, and ionic reactions, alginate can create a crosslinked structure among the G groups in the chains with different metal ions, resulting in the formation of a polymeric film [7]. As stated, the calcium ion is the most commonly used ion in the healthcare field Owing to the availability of the free electron, sodium ions can only bind with one carboxyl group in the alginate chain, whilst the calcium ion is able to react with two carboxyl groups that originate from distinct polymeric chains. To exchange sodium ions into calcium ions, sodium alginate solution should be mixed with another solution that contains calcium ions [1]. As mentioned in the same work, a G block will offer a stronger degradable gel, wherein by increasing the amount of G blocks the gel strength will be enhanced. Therefore, an alginate that is rich in G blocks will produce gels that are substantially stronger than those formed by alginate that is rich in M blocks. Additionally, it has been proved that monovalent metal-ion-bonded alginates can produce soluble salts, whilst alginates that have been bonded with multivalent cations, with the exception of magnesium ions, will form gels or precipitation. Hence, the ability of alginates to produce hydrogels is based on their affinities for different cations and their selectivity of ion binding. According to the work from Łętocha et al. [1], the concentration of polyvalent ions and alginic acid in the mixture will also affect the mechanical characteristics of the gels produced.

Recent studies have reported the development of ultrapure-alginate-derived biomaterials for biomedical and pharmaceutical applications. Owing to their high guluronic acid content and low endotoxin levels, these ultrapure alginates allow tailoring of properties and enable sensitive cell encapsulation. They have been processed into various material formats including hydrogels, fibers, films, and microcapsules. These ultrapure alginate materials present advantages over standard alginates, especially for in vivo usage where biocompatibility and precise control are critical [8].

Furthermore, alginates have been utilized to develop novel biomaterials for wound healing and anti-adhesion applications [9]. Alginate dressings containing antimicrobial agents promote healing by preventing infection. Adhesion barriers made from alginate hydrogels minimize scar formation after surgery. The biocompatibility and gelling properties of ultrapure alginates allow fabrication of bioactive patches, films, and gels to serve as next-generation wound healing and anti-adhesive materials [10].

This article reviews recent studies on the fabrication, characterization, and in vitro evaluation of various alginate-based particulate systems for delivery of drugs and nutraceuticals.

## 2. Applications of Alginate

**Figure 1:**
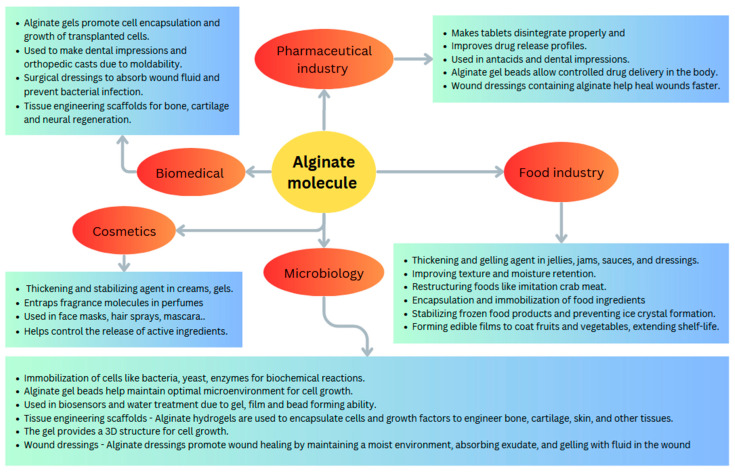
Alginate applications across a wide range of disciplines.

Alginate finds numerous applications across a wide range of disciplines due to its gelling, stabilizing, and encapsulating properties. In the biomedical field, alginate is extensively used for tissue engineering, drug delivery, wound healing, dental impressions, and probiotic encapsulation (Figure 1) [11]. Alginate hydrogels serve as scaffolds for 3D cell cultures and the engineering of tissues such as bone, cartilage, and skin. The porosity and tunable degradation of alginate gels promote cell growth, differentiation, and vascularization [12]. In pharmaceutical applications, alginate enables targeted oral drug delivery and controlled release profiles in the gastrointestinal tract. Alginate dressings absorb exudate, maintain a moist environment, and accelerate wound healing [13]. The non-toxicity and moldability of alginate also allow its use as dental impressions for braces, dentures, and oral surgeries. Alginate beads can encapsulate probiotic bacteria and maintain their viability in storage and during GI transit [14].

In food science, alginate acts as a stabilizer, thickener, gelling agent, and edible coating material. It is used to create novel textures and shapes like spheres that pop in the mouth. Alginate coatings serve as moisture barriers and oxygen barriers to improve the quality and extend the shelf-life of fruits, vegetables, and meat products [15]. In cosmetics, alginate stabilizes emulsions and provides desirable rheological properties in lotions, creams, and other personal care products [16]. In microbiology, alginate immobilization matrices are important for cell cultures and bioprocesses. The applications of alginate continue to grow with emerging fields like 3D bioprinting using alginate-based bioinks. With its versatility, biocompatibility, and beneficial physicochemical properties, alginate will continue to enable innovations across diverse disciplines [17].

**Figure 2:**
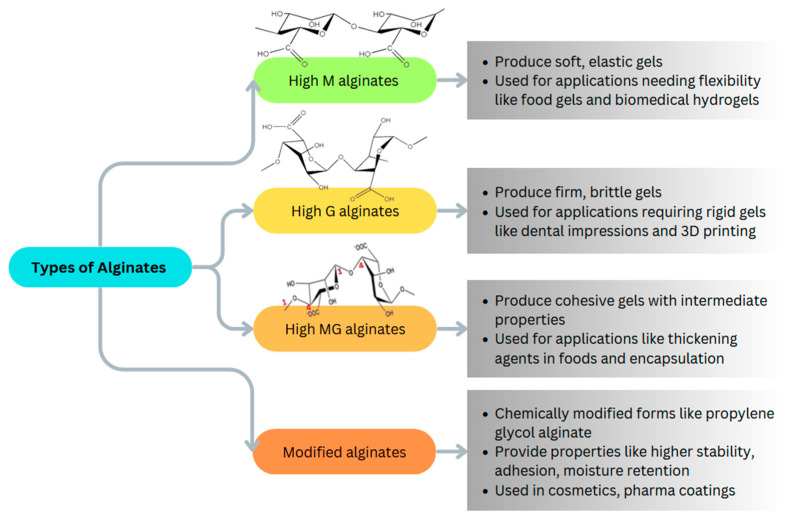
Different types of alginates and their properties.

There are various types of alginates and their properties presented in Figure 2. For example, ultrapure alginate, high-M alginate, high-G alginate, high-MG alginate, and alginate derivatives including furfuryl conjugate alginate and sodium alginate [18].

Alginates copolymers contain blocks of (1,4)-linked β-D-mannuronate (M) and α-L-guluronate (G) residues. The composition and sequential arrangement of these M and G blocks impart different physicochemical properties that make various types of alginates suitable for specific applications [19].

High-G alginates contain a greater proportion of contiguous G blocks. They readily form rigid, brittle gels upon binding with divalent cations like calcium due to the buckling of G blocks into the characteristic egg-box junctions. High-G alginates are ideal for applications necessitating firm gels with high stability like dental impressions, tissue engineering scaffolds, and 3D bioprinting [20]. 

High-M alginates possess predominantly flexible M blocks with fewer rigid G-block junction zones. This results in softer, more elastic gels. High-M alginates are widely used as thickeners and stabilizers in foods and as hydrogel matrices in pharmaceutical formulations and biomedical engineering [21].

High-MG alginates contain alternating sequences of M blocks and G blocks. They form cohesive, deformable gels with intermediate strength and stiffness. Their viscosity-enhancing and encapsulating abilities make high-MG alginates well-suited for products like salad dressings, sauces, and probiotic encapsulation [22].

Chemically or enzymatically modified alginates display tailored functionality via changes in molecular weight, hydrophobicity, adhesion, and degradation. Propylene glycol alginate has increased stability against acids and calcium chelators. Modified alginates expand the utility of alginates in cosmetics, coatings, and specialized drug delivery systems [23].

Advantages and Disadvantages of Each Type of Alginate

The distinct composition and sequencing of mannuronate (M) and guluronate (G) blocks in different alginate types confer specific advantages and limitations for various industrial and biomedical applications (Table 1) [24].

High-G alginates, with abundant G blocks, readily form stiff gels with high stability and strength via egg-box junctions between G blocks and divalent cations like calcium. This provides excellent shape fidelity for dental impressions and dimensional stability for 3D-printed constructs. However, the rigid nature of high-G gels can impair cell viability and tissue integration in more delicate biomedical applications [25]. 

Conversely, high-M alginates with dominant flexible M blocks produce soft, elastic gels. While advantageous for gentle encapsulation of drugs, proteins, or probiotics, high-M gels lack the stability required for other uses like food gels and scaffolds [20]. 

High-MG alginates offer an intermediate cohesive gel matrix which is moderately deformable yet shape-retaining. This balance suits viscosification in foods and microbeads for drug delivery. But high-MG alginates lack either the robust rigidity of high-G gels or the pliant biocompatibility of high-M gels [26].

Chemical or enzymatic modification can enhance alginate properties like stability, moisture retention, and adhesiveness. However, this may diminish advantageous innate features of natural alginates. Discerning selection of the appropriate alginate type enables exploiting their assets while mitigating shortcomings for a given application [27].

Thus, comprehension of the structure–function relationship of alginate types is key to matching their distinct mechanical and rheological profiles to the specific demands of diverse usage contexts spanning foods, pharmaceutics, coatings, 3D printing, and biomedical engineering [22].

The flow properties of alginate are critical considerations influencing its utility across industrial and biomedical applications, particularly in formulations for drug delivery. These rheological characteristics derive from the molecular weight and modifications made to the alginate polymer [11].

Native alginates extracted from brown algae form viscous polymer solutions exhibiting non-Newtonian, pseudoplastic flow behavior. Their apparent viscosity decreases with increasing shear rate. Molecular weight is a key determinant of alginate solution viscosity, with higher-molecular-weight alginates demonstrating greater viscous properties but reduced solubility. Controlled degradation via oxidative or hydrolytic means can lower molecular weight and viscosity when required [28].

Divalent cations like calcium that bind to alginate can significantly influence its flow properties. At sufficient concentrations, ionic crosslinking induces gelation, conferring shape retention. Below the gel point, ionic interactions increase viscosity in a shear-thinning manner. This allows tuning flow properties for liquid formulations [20].

Chemical modifications like carboxylation and amidation alter alginate viscosity through changes in molecular geometry, aggregation, and hydrogen bonding. Propylene glycol alginate displays lower viscosity due to disrupted polymer–solvent interactions. Hydrophobically modified alginates demonstrate shear-thinning viscosity desirable for controlled drug release [29].

### 2.1. Alginates in the Market

Alginate biopolymer has been incorporated into a wide array of commercially available products across the food, pharmaceutical, medical, and industrial sectors (Table 2). Key manufacturers have invested in scaling up production and expanding alginate’s commercial reach globally (Table 2). In pharmaceuticals, Gaviscon antacid by Bayer uses alginate raft formulations. NovaMatrix by FMC BioPolymer provides alginate for drug delivery applications. The alginate market is expected to reach USD 402.71 million in 2023 and is projected to exhibit a CAGR of 4.1%. Key drivers include growing adoption in food and biomedical sectors. Europe currently accounts for the largest share of the alginate market [30].

### 2.2. Application of Alginate in the Drug Delivery System

Alginate has become one of the most popular polymers used in the pharmaceutical industries for encapsulating drugs as a drug delivery system. Owing to its affordability, availability, and the ability to form hydrogels, alginate is shown to be more advantageous than the synthetic polymers. This notwithstanding, it has better mucoadhesion strength than the other polymers, such as chitosan, polylactic acid, and polystyrene [31]. Its bioadhesive profile would be useful as a mucosal drug delivery carrier to the gastrointestinal tract (GIT) and nasopharynx by prolonging the residence duration of the drug at the target site, and thus improving their therapeutic effectiveness [2,4]. A variety of pharmaceutical uses are also made possible by the least invasive oral administration of alginate gels. Owing to the acidic nature of the alginate, it is preferred to be used as a biodegradable polymer material as mentioned by Reddy [5]. At the same time, the highly acidic property also enables it to form gels rapidly, particularly when the calcium ions are present. 

Furthermore, numerous investigations have demonstrated that alginates, particularly those cross-linked with sodium or calcium, are not hazardous to cells and also do not cause eye or skin irritation. Therefore, it is commonly employed in various biomedical and pharmaceutical applications, for instance, in the microencapsulation of various active ingredients, with minimal adverse events [1]. In addition to this, alginate also offers some other significant advantages in terms of its biocompatibility, biostability, bioavailability, and environmentally friendly profile [31]. The hydrophilic property of alginates is also deemed to be particularly promising for therapeutic applications. As mentioned by Reddy (2021), alginates have a number of beneficial characteristics, including the capacity to form films, responsiveness to changes in pH, gelling capacity, and ionic crosslinking; therefore, they have drawn a lot of interest from the cosmetics and healthcare sectors over the past several decades [5]. 

Alginate-based drug delivery systems have numerous pharmaceutical applications and may be made into the form of microbeads, nanoparticles, and others. The novel use of alginate in the pharmaceutical industry is to create a system that can adjust drug release in response to physiological demands, such as a pH-sensitive drug release system [4]. Alginate does not need high temperature in the preparation of microbeads; at the same time, it can also boost the stability and effectiveness of the drug delivery [1]. According to the paper from Iravani and Varma [31], alginates are used in microencapsulation for controlled drug release systems for cancer therapy, in which it has been shown that alginate-based system can strengthen the targeting capabilities of the active ingredient and also improve the drug’s bioavailability, pharmacokinetic profile, and bio-clearance, with minimal toxicity and high efficacy. 

It has been demonstrated that alginate-based nanogels can improve the efficacy of *Artemisia ciniformis* extract in inducing apoptosis and inhibiting cell growth as compared with free extract [31]. According to the paper from Azad et al. (2021) [2], the peppermint-oil-loaded alginate microbeads may also enhance the drug delivery without causing any side effects. As stated in the paper from Gonçalves and coworkers [32], the sustained release of doxorubicin can also be achieved with a good encapsulation efficacy of around 80% in the form of alginate-based nanohybrids in a pH-sensitive manner. Controlled release of theophylline was also achieved by developing calcium-ion-crosslinked alginate microspheres [3]. 

In addition to this, the stability of curcumin diglutaric acid was found to be improved by encapsulating it in alginate-based nanoparticles through the oil-in-water (O/W) emulsification and ionotropic gelification method. Its drug release is also controlled and prolonged under GIT conditions, thus improving its cellular uptake by cancer cells and its anti-cancer activity [33]. By incorporating rifampicin in alginate-based nanoparticles, its redispersibility in water is improved, leading to an increase in its drug concentration in the target area and further improving its therapeutic effects. Therefore, it is deemed as one of the potential approaches to deliver drugs through a pulmonary route efficiently [34]. As mentioned in the study from Cavalu and the coworkers [32], encapsulation of selenium in alginate-based microspheres will achieve a pH-sensitive controlled drug release profile, in which drug release in gastric conditions will be minimized while its duration of action in intestinal conditions will be prolonged. 

According to the study from Freitas et al. [35], sustained release of ibuprofen will be achieved by encapsulating it in a microparticle with high alginate content. Subsequently, it will improve its therapeutic effects and patients’ compliance. The best encapsulation efficiency of the drug is also seen when the alginate content is high [35]. In addition, alginate-based cyclosporine-A-loaded microparticles were shown to have a good drug encapsulation efficiency, up to approximately 77% [36]. Controlled drug release is also achieved with this formulation, where minimal drug release is seen in stomach and small intestine conditions while a major portion of the drug is released under simulated colon conditions. Therefore, it is indicated as a promising method to deliver the drug for ulcerative colitis treatment [36]. In this context, alginate-based drug delivery systems are becoming more and more popular, especially for cancer treatment.

Table 3 summarizes recent research from 2017–2022 on the development of alginate-based encapsulation systems for delivering drugs and nutraceuticals. The studies cover a diverse range of particulate systems including microspheres, nanoparticles, microcapsules, nanogels, micelles, microfibers, and composites/scaffolds fabricated using various techniques like ionic gelation, emulsification, freeze drying, coacervation, etc. A wide variety of bioactive agents have been encapsulated in alginate particles, including antibiotics (vancomycin), antidiabetics (metformin), non-steroidal anti-inflammatories (ibuprofen), chemotherapeutics (5-fluorouracil, doxorubicin), nutraceuticals (curcumin, green tea polyphenols, astaxanthin, buriti oil), and antimicrobials (selenium). The alginate concentration used ranges from 0.5% to 4% *w*/*v*. The primary targets for drug delivery are the gastrointestinal tract (oral, intestinal, colonic) and topical (wounds, skin, mucosa). Some studies focused on targeted delivery to cancerous tissues like breast, prostate, lung, and colorectal cancers. Key findings from the research demonstrate the sustained and controlled release capabilities of alginate particles. Most formulations displayed reduced burst release and extended drug release ranging from hours to weeks compared to free drugs. Alginate encapsulation enhanced the stability and bioavailability of nutraceuticals like curcumin and astaxanthin in GI conditions. The particles improved cellular uptake and cytotoxicity of anticancer drugs against tumor cells. The systems also reduced drug toxicity by preventing systemic absorption. Wound healing efficacy was enhanced for topical delivery systems like alginate hydrogels, sponges, films, and nanofibers loaded with drugs like curcumin. These composites showed sustained drug release, mucoadhesion, improved mechanical strength, and high fluid absorbency ideal for wound dressings.

## 3. Microspheres and Nanoparticles

Alginate microspheres and nanoparticles have garnered substantial research interest for the oral administration of pharmaceuticals and nutraceuticals. The oral route remains the most preferred means of drug delivery due to its convenience, non-invasiveness, and cost-effectiveness. However, the extreme pH conditions and enzymatic barriers of the gastrointestinal (GI) tract pose challenges for the delivery of labile drugs and bioactives. In this regard, alginate-based micro/nanoparticulates provide a promising approach to protect encapsulated cargo and achieve controlled release kinetics tailored to GI transit.

Maestrelli et al. [37] adopted a freeze-drying method to fabricate alginate microspheres encapsulating metformin, an anti-diabetic drug. In vitro release kinetics demonstrated reduced burst release and sustained metformin release from the alginate microspheres in simulated intestinal fluid compared to gastric fluid. This indicates the microspheres can prevent drug release in the stomach’s acidic conditions while allowing controlled release in the neutral pH of the intestines. Oral administration of the metformin-loaded microspheres to diabetic rats showed excellent glycemic control for up to 8 h, significantly longer than free metformin. Thus, the alginate microspheres enhanced the therapeutic efficacy of metformin, underscoring the value of this encapsulation technology.

Ionotropic gelation, based on alginate crosslinking with divalent cations, provides a simple and mild method for forming alginate microparticles. Shahnia [38] utilized this approach to develop curcumin-loaded alginate microparticles, which displayed sustained release kinetics and improved the wound healing potency of curcumin in rats. The study highlights the feasibility of ionotropic gelation for fabricating alginate microparticulates for drug delivery applications.

In addition to microspheres, alginate nanoparticles have also been extensively researched for oral drug delivery. Manatunga et al. [39] synthesized curcumin-loaded alginate nanoparticles using a co-precipitation technique. In vitro analyses revealed sustained curcumin release from the nanoparticles compared to free curcumin. Oral administration in rats showed a 5-fold increase in the bioavailability of the encapsulated curcumin versus free curcumin. The study underscores the potential of alginate nanoparticles to enhance the bioavailability of poorly soluble drugs like curcumin.

Cavalu et al. [40] developed alginate microspheres containing selenium by a combination of crosslinking and ionotropic gelation. In vitro release profiles showed negligible selenium release in simulated gastric fluid but sustained release in intestinal fluid. This indicates the feasibility of using alginate microspheres for site-specific intestinal delivery. Moreover, oral administration of the selenium-loaded microspheres in rats demonstrated their safety and efficacy in supplementing dietary selenium.

Alginate nanoparticles have also been applied toward the targeted delivery of chemotherapeutics. Saralkar and Dash [42] prepared curcumin-loaded nanoparticles using emulsification and crosslinking. The nanoparticles displayed enhanced cellular uptake and cytotoxicity against prostate cancer cells. Zhang et al. [43] developed 5-fluorouracil-loaded alginate nanoparticles by freeze drying. The nanoparticles exhibited colon-specific drug release and reduced drug toxicity in rats. Shen et al. [44] fabricated doxorubicin-loaded alginate nanoparticles using layer-by-layer coating. The nanoparticles showed improved cellular uptake and tumor growth inhibition compared to free doxorubicin in drug-resistant breast cancer cells.

Several studies have explored alginate nanoformulations for combination drug delivery. Song et al. [45] prepared curcumin-loaded alginate nanoparticles using co-precipitation. The nanoparticles displayed sustained release and enhanced cytotoxicity against breast cancer cells. Hosseinifar et al. [46] developed 5-fluorouracil-loaded alginate nanogels using emulsification and crosslinking. The nanogels showed colon-specific drug release, rapid cellular uptake, and high cytotoxicity against colon cancer cells.

## 4. Microcapsules and Microbeads

Alginate microcapsules and microbeads have garnered attention for oral delivery applications focused on nutraceuticals, antibiotics, and anti-inflammatories. Microencapsulation of oils and antioxidants in alginate matrices aims to improve stability and enable controlled release kinetics tailored to nutrient absorption profiles. Furthermore, alginate microparticulates are well-suited for tailored and sustained release of drugs in the gastrointestinal tract. Additional coatings on alginate microcapsules provide opportunities to further modify drug release and improve stability.

Lemos et al. [41] prepared alginate microcapsules containing buriti oil, valued for its high carotenoid content, using complex coacervation with gelatin. The microcapsules demonstrated an excellent encapsulation efficiency of 80%, indicating the suitability of complex coacervation for encapsulating oils. Protection of oils and antioxidants by an alginate matrix can enhance their stability against environmental factors like light, oxygen, and elevated temperatures during processing, storage, and gastric transit. Controlled release in the intestines can synchronize the availability of lipophilic nutrients with lipid digestion and absorption. However, in vitro release kinetics of buriti oil from the alginate microcapsules were not investigated in this study and remain an area for further research.

Astaxanthin is a carotenoid antioxidant whose encapsulation can also improve stability and bioavailability. Li et al. [48] developed astaxanthin-oleoresin-loaded alginate microcapsules, also using complex coacervation. In vitro release studies revealed minimal release in simulated gastric fluid versus rapid release in simulated intestinal fluid, indicating suitability for targeted release in the intestines. The alginate coating improved the thermal, oxidative, and photo stability of astaxanthin. Oral administration in mice showed higher plasma levels and bioavailability of astaxanthin from the microcapsules compared to free oleoresin. Thus, the alginate microcapsules enhanced the intestinal absorption and bioavailability of astaxanthin, demonstrating their utility for delivering lipophilic nutraceuticals.

In addition to encapsulating lipids, alginate microparticulates are suitable for tailored delivery of hydrophilic drugs in the GI tract. Unagolla and Jayasuriya [47] developed vancomycin-loaded alginate microparticles using ionotropic gelation, which provided sustained antibiotic release for two weeks in vitro. This prolonged release can improve patient compliance by reducing dosing frequency and enable greater bacterial exposure to vancomycin to improve eradication. While the study did not assess antimicrobial efficacy in vivo, the findings warrant further investigation of the alginate microparticles for treating bacterial infections, particularly of the GI tract.

Alginate microparticles prepared via ionotropic gelation can also offer sustained release of drugs like non-steroidal anti-inflammatories (NSAIDs). Freitas et al. (2018) [35] investigated the effect of alginate concentration on the release of ibuprofen from alginate microparticles. Higher alginate concentrations delayed the release rate and extent of ibuprofen, demonstrating that alginate content can be leveraged to modulate drug release kinetics. The results underscore the versatility of the ionotropic gelation method and the ability of alginate microparticles to provide sustained NSAID release tailored to dosing requirements. Further in vivo assessment is required to evaluate the pharmacokinetic and pharmacodynamic advantages of the sustained release system.

Surface coatings present another strategy to tailor the release kinetics of alginate microcapsules for targeted drug delivery applications. Oshi et al. [36] developed Eudragit-S100-coated, cyclosporine-loaded alginate microparticles prepared by ionotropic gelation. Eudragit S100 dissolves at pH > 7, conferring colon-specific drug release. In vitro analyses verified negligible cyclosporine release in simulated gastric and intestinal fluids, but sustained release in simulated colonic fluid. This indicates the coating can prevent systemic absorption and provide colon-targeted release to treat ulcerative colitis, although in vivo verification is needed.

Frenț et al. [68] also adopted a coating approach, fabricating quercetin-loaded alginate microspheres with complex coacervation using chitosan. The chitosan coating enhanced quercetin’s stability against heat and oxidation, while also modifying its release. In vitro analyses showed low quercetin release in simulated gastric conditions but sustained release at intestinal pH, indicating the microspheres can shield it from gastric degradation and provide controlled intestinal delivery. Furthermore, the coating prevented enzymatic degradation of quercetin by alpha-amylase.

## 5. Microgels, Micelles, and Nanofibers

Novel alginate nanostructures such as microgels, micelles, and nanofibers have emerged as versatile drug delivery platforms. Their nanoscale features and high surface area to volume ratio impart advantages including improved drug loading, controlled release kinetics, and cell penetration. Furthermore, their injectable nature and mucoadhesive properties make these nanostructures well-suited for localized drug delivery applications.

Sorasitthiyanukarn et al. [33] prepared self-assembled curcumin-loaded alginate micelles through ionic gelation between alginate and curcumin diglutamate. In vitro release studies showed sustained curcumin release from the micelles for up to 5 h in physiological intestinal conditions. Conventional curcumin micelles suffer from rapid drug release, making these alginate micelles advantageous for prolonged cargo delivery. However, in vivo studies are warranted to evaluate their therapeutic performance. An innovative approach was adopted by Lachowicz et al. [50] who developed curcumin-loaded nanomicelles through self-assembly of alginate grafted with pluronic polymer chains. The pluronic chain confers temperature-responsiveness to the micelles for targeted drug release. The nanomicelles demonstrated excellent colloidal stability with no aggregation or hemolysis. Rapid cellular uptake within 1 h was observed in vitro, highlighting the permeability advantages of the nanosized micelles. This study demonstrates the potential of novel polymer–alginate conjugates to engineer nanostructures tailored for drug delivery. Alginate microgels are also gaining interest as injectable sustained release depots. Chen et al. [49] synthesized novel pH-sensitive microgels via self-assembly of alginate and green tea polyphenol. The microgels demonstrated pH-triggered drug release, e.g., negligible release under gastric conditions but sustained cargo release at intestinal pH. This pH-responsive release can minimize premature drug leakage in the stomach and achieve controlled delivery in the intestines. Though not yet assessed in vivo, these microgels show promise as smart oral drug carriers.

Wound healing represents a niche application where alginate nanofibers and microfibers have displayed utility as localized and sustained release drug depots. Gnanamangai et al. [51] developed curcumin-loaded alginate nanofibers using a freeze gelation technique. The nanofibers exhibited enhanced curcumin stability and showed excellent anticancer effects by inhibiting proliferation of lung cancer cells, while being biocompatible with normal lung fibroblasts. This demonstrates their potential for localized and sustained chemotherapy to treat lung tumors. Sharma et al. [60] fabricated curcumin-alginate microfibers using ionotropic gelation and showed their efficacy as wound dressings. The microfibers enabled sustained curcumin release over 72 h and accelerated wound closure in rats, highlighting their promise as treatments for chronic wounds.

## 6. Composite and Scaffold Systems

Composite systems incorporating alginate with other polymers have shown promise for topical and tissue-engineering applications. Combining alginate with biomaterials like chitosan and cellulose can modify the physicochemical and release properties of the delivery platform to achieve localized and sustained drug delivery for improved therapeutic outcomes. Furthermore, developing alginate-based scaffolds offers new horizons in tissue engineering and regenerative medicine.

Chiaoprakobkij et al. [59] prepared curcumin-loaded films by mechanical blending of alginate and chitosan followed by solution casting. In vitro studies showed potent dose-dependent anticancer effects of the composite films against oral cancer cells, indicating sustained curcumin release. Mucoadhesion testing verified an optimum 30–36 min adherence time for porcine oral mucosa under simulated salivary conditions. This demonstrates potential of the films as localized oral drug delivery to treat lesions and tumors. Further in vivo assessment of the therapeutic efficacy and safety of these composite films is warranted.

Mobaraki et al. [66] developed an innovative approach by incorporating curcumin-loaded alginate nanoparticles into chitosan scaffolds prepared by freeze drying. In vitro analysis showed curcumin was released in a controlled manner from the scaffolds for up to 14 days. The nanoparticle-in-scaffold platform combined the drug carrier capabilities of alginate with the structural and release advantages of chitosan scaffolds. However, the therapeutic efficacy of these scaffolds remains to be evaluated for wound healing or tissue-engineering applications. This study provides a proof-of-concept for composite scaffold systems with alginate nanoparticles. Alginate sponges fabricated via freeze drying have also garnered attention for wound healing applications, owing to their highly porous structure enabling fluid absorption and drug delivery. Zhao et al. [56] developed curcumin-loaded alginate sponges and demonstrated their efficacy in diabetic rats. The sponges showed improved curcumin loading and twice the release of a carbopol analogue. Topical application enhanced wound closure compared to controls, indicating accelerated healing mediated by sustained curcumin delivery. Further assessment of their mechanical properties, cytocompatibility, and effects on wound microbiota would aid clinical translation. Nevertheless, this study highlights the promise of alginate sponges as bioactive wound dressings.

Guadarrama-Acevedo et al. [57] presented an innovative approach by incorporating curcumin-loaded alginate nanoparticles into polymeric wound dressings. The nanocomposite dressing provided controlled curcumin release for up to 72 h and showed adequate mechanical strength, flexibility, and fluid handling properties. In vivo evaluation in rats demonstrated the sustained analgesic and anti-inflammatory effects of curcumin released from the dressings. The study provides an important proof-of-concept for multifunctional nanocomposite dressings with drug delivery capabilities. Further research should evaluate their effects on infection control and healing outcomes in chronic wounds.

## 7. Future Directions and Challenges

Alginate-based biomaterials have demonstrated versatile properties enabling engineering of diverse drug delivery systems across therapeutic areas. Proof-of-concept studies highlight the promise of alginate for controlled and localized delivery. However, further innovation in alginate composition and preparation methods is imperative to optimize delivery kinetics and stability for specific drug cargos and routes. Tailoring crosslinking density, molecular weight, and formulation parameters can significantly influence encapsulation, release kinetics, and stability. Mathematical modeling approaches leveraging artificial intelligence could expedite rational selection of these parameters to achieve target product profiles. Moreover, quality-by-design-based manufacturing processes must be developed to enable scalable and consistent production with batch-to-batch consistency. 

More rigorous in vivo testing is critical to evaluate the pharmacokinetic advantages, therapeutic efficacy, tissue compatibility, and toxicity profiles of developed systems. While many studies demonstrate controlled release in vitro, testing in animal models is essential to assess clinical performance and bioavailability enhancement. Safety and biocompatibility remain paramount considerations. Exploring combinatorial strategies like alginate composites with polymers such as chitosan and Eudragit could augment functionality for cutting-edge applications. Incorporating environment-responsive elements could add triggers for on-demand cargo release in response to biochemical stimuli. 

Alginate-integrated 3D printing has disruptive potential to transition from simple depots to architecturally complex, personalized constructs with defined shapes, porosities, and spatial drug distribution. A 3D-printed alginate scaffold with tailored release capabilities could better recapitulate native tissue environments. Composite printing with bioinks can produce multifunctional platforms combining the merits of alginate and other biomaterials. Ingestible electronic systems incorporating alginate particles enable real-time monitoring and targeted on-demand drug release, paving the way for smart delivery systems.

Emerging nanomedicine advances also offer opportunities for innovative alginate nanoconstructs. Alginate nanoparticles and micelles confer advantages like rapid cellular permeation due to nanoscale dimensions. Combining alginate with nanomaterials such as carbon nanotubes, graphene, quantum dots, and gold nanoparticles could impart new sensing, targeting, and stimulus-responsive properties. However, toxicity concerns must be addressed.

For cancer therapies, alginate systems may enable improved delivery of emerging biotherapeutics like siRNA, mRNA, and antibodies. Sustained release of proteins, antibodies, and cellular therapeutics by alginate matrices may enhance pharmacotherapy. Targeting cancer-associated fibroblasts with local anti-fibrotic agent delivery using alginate depots could combat metastasis. 

However, alginate’s lack of degradability has constrained its adoption for applications requiring material resorption. In wound dressings, the non-degrading matrix necessitates removal after healing, unlike digestible materials. In tissue engineering, inert alginate scaffolds require eventual surgical retrieval, unlike degradable scaffolds remodeled by cells. For cell delivery, alginate vehicle extraction is needed. The non-degradability also limits suitability for drug delivery systems expected to break down in vivo after cargo release.

Strategies are being developed to impart degradability like oxidation, hydrolysis, chemical/enzymatic scission, and blends with degradable polymers to introduce tunable degradation rates. Advances rendering alginate susceptible to cell-mediated, hydrolytic, or enzymatic degradation will expand utility across modalities needing eventual resorption.

Technical challenges remain regarding large-scale GMP production capabilities and regulatory approval to demonstrate safety and efficacy. Intellectual property barriers may need navigating to avoid infringement issues. Alginate’s promise can only be realized through continued innovation addressing limitations, coupled with strategies enabling real-world impact. Alginate-based drug delivery systems hold tremendous potential for advancing pharmaceutical sciences.

## 8. Conclusions

Alginate has emerged as a versatile biopolymer material for pharmaceutical applications owing to its biocompatibility, relatively low cost, ease of chemical modification, and mild gelation under physiological conditions. A myriad of drug encapsulation approaches has been investigated, including microspheres, nanoparticles, micelles, microgels, and composites fabricated by techniques such as ionic gelation, emulsification, spray drying, and freeze drying. In vitro studies have demonstrated excellent capabilities for tailored and sustained release of both hydrophilic and hydrophobic drugs. Alginate microparticles provide gastric protection and colon-targeted release of orally delivered drugs while alginate nanoparticles and microgels show promise for enhancing stability and drug bioavailability. Alginate-based wound dressings have also found extensive use. While in vivo animal studies have shown positive results, more research is needed to assess clinical safety and efficacy in humans before alginate systems can gain pharmaceutical approval. Nonetheless, the versatility of alginate encapsulation methods coupled with emerging approaches to engineer smart, targeted, and personalized delivery continues to expand the horizons for alginate biomaterials across diverse biomedical applications.

## Figures and Tables

**Table 1:** The advantages and disadvantages of different alginate types for various applications.

Alginate Type	Applications	Advantages	Disadvantages
High-G alginates	Dental impressions, 3D printing, tissue engineering	Strong rigid gels, high stability, excellent printability	Brittle gels, low biocompatibility
High-M alginates	Food gels, biomedical hydrogels	Soft elastic gels, good biocompatibility	Weak gels, low stability
High-MG alginates	Thickeners, encapsulation	Cohesive gels, viscosity enhancement	Intermediate strength and stability
Modified alginates	Cosmetics, coatings, specialized delivery	Enhanced stability, adhesion, moisture retention	Can lose intrinsic properties of natural alginate

**Table 2:** Some commercially available products containing alginate.

Product	Manufacturer	Use	Alginate Type
NovaMatrix	FMC BioPolymer	Pharmaceutical excipient for oral drug delivery	High-G sodium alginate
Kaltostat	ConvaTec	Wound dressing with hemostatic properties	Calcium alginate
Gaviscon	Reckitt	Antacid formulation	Sodium alginate

**Table 3:** Summary of alginate-based encapsulation fabrication techniques for drug delivery.

Types of Particle	Formulation Technique	Active Pharmaceutical Ingredient	Concentration of Alginate	Target System	Findings	Reference
Microsphere	Freeze drying	Metformin	-	Intestine	Controlled drug release in gastric conditions and sustained drug release in intestinal conditions are achieved with this formulation. This notwithstanding, the therapeutic effect of metformin is also enhanced. Therefore, this formulation is deemed as a potential approach to deliver metformin for a prolonged duration of action.	Maestrelli et al., [37]
Microparticle	Ionotropic gelation	Curcumin	0.63 mg/mL	Topical	The fabricated microparticles were shown to have an enhanced wound healing effect with a drug encapsulation efficiency of 75%. Moreover, a sustained drug release mechanism is also achieved with this alginate-based formulation, hence proving the potential use of this biodegradable formulation to deliver curcumin topically.	Shahnia, [38]
Nanoparticle	Co-precipitation approach	Curcumin	40%	GIT	This alginate formulation exhibits an excellent sustained drug release profile in a pH-sensitive manner. A prolonged duration of action of the drug is also observed. With its good encapsulation efficiency and the drug release profile, it is indicated as one of the potential methods to achieve targeted drug delivery.	Manatunga et al., [39]
Microsphere	Cross-linking and ionotropic gelation	Selenium	1.5% *w*/*w*	Intestine	Minimal drug release in gastric conditions is observed with this formulation, while the release duration and duration of action in intestinal conditions are prolonged, hence indicating a pH-sensitive drug release profile. Therefore, this alginate-based formulation is deemed as one of the potential approaches to deliver selenium in the intestine.	Cavalu et al., [40]
Microcapsule	Complex coacervation	Buriti oil	1% *w*/*w*	-	Encapsulation efficiency of approximately 80% is achieved with this formulation.	Lemos et al., [41]
Nanoparticle	Emulsification and cross-linking process	Curcumin	0.6 mg/mL	Prostate	Slow drug release and high cellular uptake of curcumin are demonstrated with this formulation. Moreover, curcumin’s cytotoxic action towards prostate cancer cells is also observed with the absence of hemolysis after the administration, proving its safety for human use.	Saralkar & Dash, [42]
Nanoparticle	Freeze drying	5-fluorouracil	2.5% *w*/*v*	Intestine (Colon)	This alginate-based formulation exhibits a colon-targeted drug release profile with a reduced drug toxicity. Therefore, it is deemed as a potential approach to deliver the drug for a colon-targeted treatment.	Zhang et al., [43]
Microcapsule	Layer-by-layer (LbL) technique	Doxorubicin	-	Breast	This formulation revealed a better cellular uptake and cytotoxic action against the breast cancer cells as compared with the free drug. Prolonged retention time in targeted areas has also been demonstrated. Therefore, it is deemed as a significant drug delivery method for treating drug-resistant breast cancer.	Shen et al., [44]
Nanoparticle	Co-precipitation OR layer-by-layer coating	Curcumin	20 mg/mL	Breast	This alginate-based formulation has demonstrated a sustained drug release profile, along with an enhanced cellular uptake efficiency, as compared to the free drug. Its cytotoxic effect against cancer cells is also improved. Hence, it is one of the most promising approaches to deliver the drug for cancer treatment.	Song et al., [45]
Nanogel	Cross-linking/emulsification	5-Fluorouracil	1.8% *w*/*w*	Intestine (Colon)	The prepared hydrogels are found to be cytocompatible with an encapsulation efficiency of up to 82%, where the drugs are able to be released under conditions that are similar to intravascular pressure. In addition, this formulation can also be rapidly uptaken by the colon cell line with high cellular accumulation as compared to the free drug. Therefore, it is considered as a promising approach to deliver the drug for colon cancer therapy.	Hosseinifar et al., [46]
Microparticle	Ionotropic gelation (coacervation) technique	Vancomycin	1–2% *w*/*v*	Intestine	A controlled drug release profile with an average release rate of 22 μg/d for 2 weeks is observed in this formulation.	Unagolla and Jayasuriya, [47]
Microcapsule	Complex coacervation technique	Astaxanthin oleoresin	0.5% *w*/*v*	Intestine	The fabricated microcapsules have shown a good encapsulation efficiency of up to 85.9%. In addition, the stability of the active ingredient against the heat, oxygen, and light is improved too, subsequently improving its bioavailability. A controlled drug release is demonstrated in intestinal conditions, where a rapid release of the drug is observed under an alkaline environment, proving that the coating material is able to resist the acidic conditions.	Li et al., [48]
Microgel sphere	-	Green tea polyphenol	2%	Bone	A controlled drug release is achieved with this formulation in a pH-sensitive manner. High trapping efficiency of approximately 92% is also observed.	Chen et al., [49]
Microparticle	Ionotropic gelation method	Ibuprofen	1–2.8% *w*/*v*	GIT	This study has proved that with high alginate content, sustained drug release profile and better drug encapsulation will be achieved.	Freitas et al., [35]
Nanoparticle	O/W emulsification and ionotropic gelification	Curcumin diglutaric acid	0.6 mg/mL	Intestine (Colon), liver, and breast	Stability under UV radiation and in the simulated gastrointestinal environment is improved with this formulation. In addition, a delayed cumulative drug release in GIT condition is achieved. Cellular uptake by colon, liver, and breast cancer cells is also improved, as well as its anti-cancer effects. Hence, it is deemed as a potential method to deliver drugs for cancer therapy.	Sorasitthiyanukarn et al., [33]
Micelle	-	Curcumin	-	-	A controlled drug release profile is observed for a prolonged period of up to 5 h under physiological conditions. Its safety for human use has been confirmed by the absence of aggregation and hemolysis of red blood cells after administration. It is also found that it can be rapidly uptaken by the cell, in which the highest concentration was observed within 1 h after the treatment. Hence, it is deemed as a safe and efficient drug delivery system for curcumin.	Lachowicz et al., [50]
Microencapsulated matrix/nanofibrous scaffold	Freeze gelation	Curcumin	-	Topical (Wound)	Excellent anti-cancer activity against cancer cells on both normal and human lung adenocarcinoma cells is observed. This formulation is also found to be stable against enzymatic degradation.	Gnanamangai et al., [51]
Microcapsule	-	*Stellaria media*	2% *w*/*v*	GIT	A good encapsulation efficiency of up to 92.47% is achieved with this formulation. Controlled drug release mechanism is also observed, where a major portion of the drug is released in intestinal conditions rather than gastric conditions.	Miere et al., [52]
Nanomicelle/microsphere	-	Dasatinib and zein-lactoferrin	-	Breast	Sustained drug release profile is achieved with this formulation. The fabricated formulation also showed excellent cytotoxic action against the breast cancer cell line. This notwithstanding, this alginate-based formulation also demonstrated a significant encapsulation efficiency.	Ragab et al., [53]
Nanoparticle	Ionotropic gelation technique	Curcumin	1%	Intestine (Colon)	An improved encapsulation efficiency of up to 95% is observed with this formulation. This formulation also demonstrated an extremely low dissolution rate in GIT conditions, in which the major portion of the drug is released in colon conditions. Additionally, the drug bioavailability is also shown to be enhanced by 5 times after the encapsulation.	Govindaraju et al., [54]
Nanoparticle	Ionic gelation technique	Curcumin	0.025% *w*/*v*	-	Drug loading efficiency up to 90% is observed with this formulation, with a sustained drug release profile.	Pornpitchanarong et al., [55]
Sponge (wound dressing)	Freeze-drying	Curcumin	1%	Topical	This formulation is shown to be able to facilitate the wound healing process by improving the drug release and water uptake profiles.	Zhao et al., [56]
Nanoparticle/wound dressing	Emulsification–diffusion method	Curcumin	4% *w*/*v*	Topical	High mechanical strength with good absorbency ability are achieved with this formulation. Additionally, controlled drug release profiles with delayed degradation are also observed.	Guadarrama-Acevedo et al., [57]
Nanoparticle	Ionic gelation method	Rifampicin	0.06% *w*/*v*	Pulmonary route	Improved therapeutic efficacy is revealed with this formulation, mainly owing to its improved redispersibility in water. Drug concentration in the targeted area is also increased, while the systemic toxicity of the drug is reduced. Therefore, it is deemed as a potential method to deliver the drug through a pulmonary route.	Scolari et al., [34]
Microparticle	Spray drying	Curcumin	1% *w*/*v*	-	A slow drug release is observed with this formulation. In addition, encapsulation efficiency up to 97.6% is also achieved.	Lucas et al., [58]
Biopolymer composite film	Mechanical blending and casting method	Curcumin	-	Topical	The prepared formulation exhibits a potent anti-cancer effect towards the oral cancer cells, whilst its mucoadhesion time for porcine mucosa is observed as 30 to 36 min under the artificial saliva conditions.	Chiaoprakobkij et al., [59]
Microfiber	Ionotropic gelation method	Curcumin	10–100%	Topical	A prolonged drug release is observed with this formulation for up to 85% in 3 d. Owing to its significant effect on wound healing, this formulation is indicated as a potential approach for wound management.	Sharma et al., [60]
Nanogel	Cross-linking process	Oxaliplatin	-	Intestine (Colorectal)	Improved anti-cancer activity towards the colorectal adenocarcinoma cell line is seen with this formulation. Therefore, it is considered as a significant approach to deliver oxaliplatin for colorectal cancer treatment.	Shad et al., [61]
Microsphere	Spray drying	Ropinirole hydrochloride	0.5% *w*/*v*	Nasal epithelium	Excellent stability profile and high drug trapping efficiency, up to 106%, are observed with this formulation.	Hussein et al., [62]
Nanosphere	Ionotropic gelification	Curcumin	-	Breast	Great drug encapsulation efficiency and delayed drug release up to 24 h are observed. Curcumin’s absorption is also improved. After the administration, the proliferation of the breast cancer cells is significantly reduced.	Afzali et al., [63]
Microbead	In situ ion-exchange followed by simple ionotropic gelation technique	Curcumin	-	Intestine	Extended release of curcumin is revealed with this alginate-based formulation, subsequently improving its bioavailability and anti-tumor effect.	Sreekanth Reddy et al., [5]
Microparticle	Ionic gelation/ eudragit S 100 coating	Cyclosporine A	-	Intestine (Colon)	This formulation has achieved a good drug encapsulation efficiency of approximately 77%. During the study, it has been found that the drug release was inhibited under stomach and small intestine conditions, whilst under a simulated colon environment, a drug dissolution followed by a sustained drug release is achieved, hence minimizing the systemic absorption and side effects. Hence, it is considered as a promising approach to deliver the drug for the treatment of ulcerative colitis.	Oshi et al., [36]
Nanoparticle/scaffold	-	Curcumin	2%	Topical	A good drug encapsulation profile is achieved with this formulation.	Mobaraki et al., [64]
Hydrogel bead	Ionotropic gelation method	Curcumin	2%	GIT	Controlled drug release is achieved with this formulation under simulated GI conditions.	Sharma et al., [65]
Nanoparticle	-	Curcumin	1% *w*/*v*	Intestine (Colon)	A good drug encapsulation efficiency up to 91% is observed in this study. Moreover, it is also found that the drug absorption by the colon cancer cells is improved with the prepared formulation, as compared to the free drug, subsequently improving its target efficiency. Therefore, this alginate-based formulation is indicated as one of the most promising approaches for treating colon cancer.	Liu et al., [66]
Hydrogel	Ionic gelation technique	Curcumin	-	Lung and breast	A better anti-cancer effect is proved with this formulation.	Torbati et al., [67]
Microsphere	Complex coacervation technique	Quercetin	0.75% *v*.*v*	GIT	High drug trapping efficiency of approximately 86% is achieved, as well as the controlled release of drugs. This notwithstanding, it is found that the thermal stability of the drug is enhanced with this formulation and, owing to the lipophobicity of the matrix, quercetin is protected from the enzymatic degradation in the GIT.	Frenț et al., [68]
Microsphere/microbead	Emulsion-templated ionic gelation	Curcumin/5-FU	3%	Breast	A pH-sensitive drug delivery system is revealed in this study. Moreover, the anti-cancer activity of the drugs are also improved.	Boddu et al., [69]

## Data Availability

Data are contained within the article.
